# Combined Treatment With CCR1-Overexpressing Mesenchymal Stem Cells and CCL7 Enhances Engraftment and Promotes the Recovery of Simulated Birth Injury-Induced Stress Urinary Incontinence in Rats

**DOI:** 10.3389/fsurg.2020.00040

**Published:** 2020-07-31

**Authors:** Hai-Hong Jiang, Ling-Xiao Ji, Hai-Yan Li, Qi-Xiang Song, Yasmeen Bano, Lei Chen, Guiming Liu, Meihao Wang

**Affiliations:** ^1^Department of Urology and Andrology, The First Affiliated Hospital of Wenzhou Medical University, Wenzhou, China; ^2^Department of Radiology, The First Affiliated Hospital of Wenzhou Medical University, Wenzhou, China; ^3^Department of Urology, Changhai Hospital, The Second Military Medical University, Shanghai, China; ^4^Department of Surgery/Urology, MetroHealth Medical Center, Case Western Reserve University, Cleveland, OH, United States

**Keywords:** stress urinary incontinence, mesenchymal stem cell, chemokine (c-c motif) ligand 7, chemokine (c-c motif) receptor 1, rat model, birth injury

## Abstract

**Objective:** To observe whether urethral injection of chemokine (c-c motif) ligand 7 (CCL7) and overexpressing CC receptor 1 (CCR1) in mesenchymal stem cells (MSCs) can promote their homing and engraftment to the injured tissue, and improve the recovery of simulated birth injury-induced stress urinary incontinence (SUI) in rats.

**Methods:** Female rats underwent a dual injury consisting of vaginal distension (VD) and pudendal nerve crush (PNC) to induce SUI. Bone marrow-derived MSCs were transduced with lentivirus carrying CCR1 (MSC-CCR1) and green fluorescent protein (GFP). Forty virgin Sprague–Dawley rats were evenly distributed into four groups: sham SUI + MSC-CCR1+CCL7, SUI + MSCs, SUI + MSC-CCR1, and SUI + MSC-CCR1+CCL7 group. The engrafted MSCs in urethra were quantified. Another three groups of rats, including sham SUI + sham MSC-CCR1+CCL7 treatment, SUI + sham MSC-CCR1+CCL7 treatment, and SUI + MSC-CCR1+CCL7 treatment group, were used to evaluate the functional recovery by testing external urethral sphincter electromyography (EUS EMG), pudendal nerve motor branch potentials (PNMBP), and leak point pressure (LPP) 1 week after injury and injection. Urethra and vagina were harvested for histological examination.

**Results:** The SUI + MSC-CCR1+CCL7 group received intravenous injection of CCR1-overexpressing MSCs and local injection of CCL7 after simulated birth injury had the most engraftment of MSCs to the injured tissues and best functional recovery from SUI compared to other groups. Histological examination showed a partial repair in the SUI + MSC-CCR1+CCL7 group.

**Conclusions:** Our study demonstrated combined treatment with CCR1-overexpressing MSCs and CCL7 can increase engraftment of MSCs and promote the functional recovery of simulated birth trauma-induced SUI in rats, which could be a new therapeutic strategy for SUI.

## Introduction

Urinary incontinence (UI) is becoming an individual and social burden with the increased aging population. More than 40% of women over 40 years old have symptoms of UI in the general population (1). Stress urinary incontinence (SUI) is the most common type of UI in women, which is related to the urethra and pelvic floor injury during childbirth (2). During the process of parturition, the vaginal walls and muscles, ligaments, and connective tissues of the pelvic floor can be compressed and overstretched (3, 4), especially at the second stage of labor with injuries of the tissue laceration and pudendal nerves (4, 5).

Mesenchymal stem cells (MSCs) are multipotent adult progenitor cells, which can migrate to the location of injury to facilitate repair and regeneration via differentiation and paracrine or autocrine mechanisms (6–8). Therefore, MSCs may play an important role in endogenous tissue repair and regeneration (9). Recently, cell-based therapies were developed for the treatment of acute injuries and degenerative diseases (10–12). Despite these progresses, there are many barriers in carrying out stem cell therapy in current practice. Engraftment and survival of the transplanted cells into the injured or ischemic area is still a problem as most of the cells are lost within hours of transplantation. Moreover, the detailed mechanisms determining these processes are still not well-understood.

Previous studies have demonstrated that chemokines play key roles in MSCs homing to sites of injury (13–16). Chemokine and chemokine receptors regulate chemotactic activities of a wide range of cell types, such as monocytes and stem cells (14, 17, 18). Several chemokines, including chemokine (c-c motif) ligand 7 (CCL7), chemokine (c-x-c motif) ligand 1 (CXCL1), and ligand 2 (CXCL2), have been shown to be upregulated in the local lower urinary tract following vaginal delivery, indicating that they may regulate the stem cell engraftment into urinary tissues after childbirth injury (19). Indeed, recent studies have confirmed that cytokine CCL7 plays a key role in the engraftment of MSCs (20, 21). Chemokine (c-c motif) receptor 1 (CCR1) is one of the receptors for CCL7 (22). Although functionally active chemokine receptors have been identified in MSCs, the expression levels are relatively low (23). On the other hand, overexpression of CCR1 in MSCs was reported to enhance their migration, survival, and engraftment after myocardium injury (24).

We hypothesize that enhancement of chemokine and chemokine axis by local injection of CCL7 and intravenous transfusion of CCR1-overexpressing MSCs can improve engraftment and survival of the MSCs into the injured tissues and promote the functional recovery after a simulated birth injury.

## Materials and Methods

The Animal Care Committee at the First Affiliated Hospital of Wenzhou Medical University approved all animal protocols and surgical procedures described below.

### VD+PNC Induced Simulated Birth Injury

**Figure 1:**
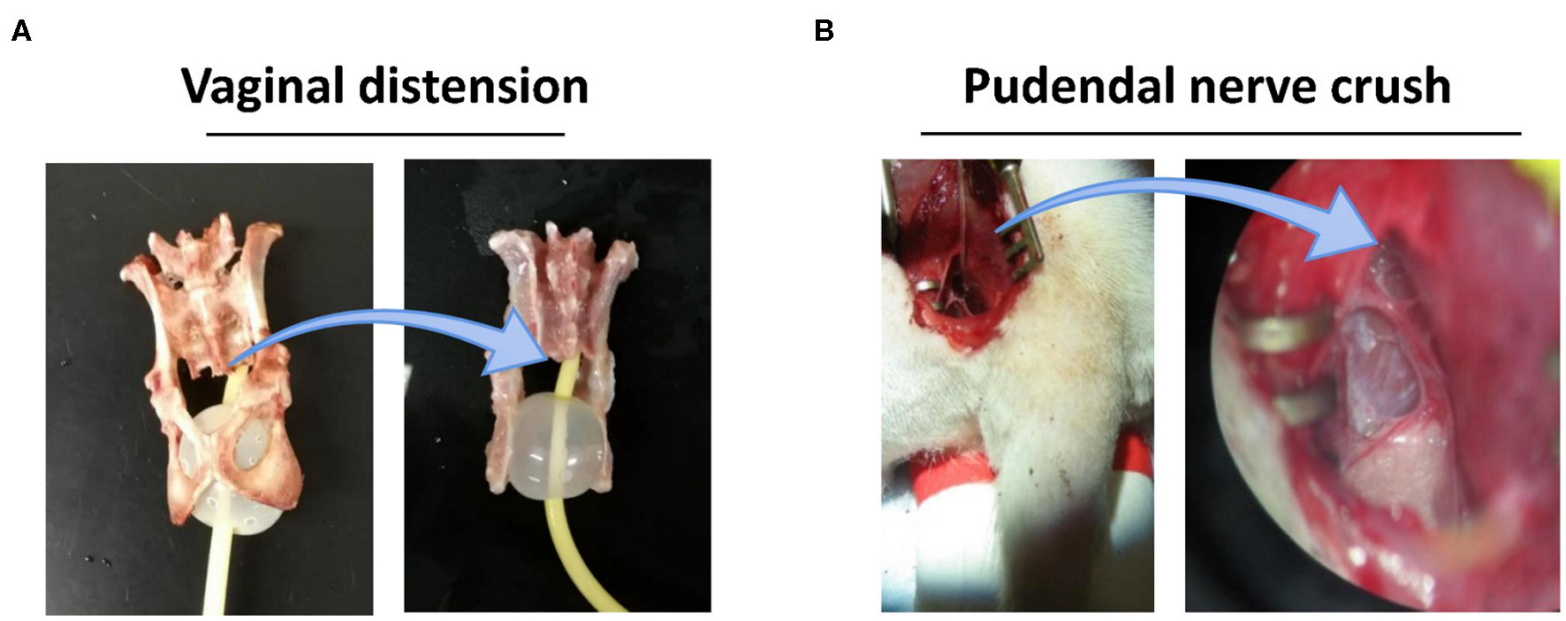
VD+PNC simulated birth injury postpartum model for stress urinary incontinence condition. **(A)** A 10F Foley catheter balloon with 3 ml water in a female rat pelvic cavity illustrates vaginal distension condition. **(B)** Dorsal approach for pudendal nerve crush after opening ischiorectal fossa under microscopic. VD, vaginal distension; PNC, pudendal nerve crush.

We have demonstrated that a dual injury consisting of vaginal distension (VD) and pudendal nerve crush (PNC) can induce a reliable model of SUI (25) Such dual-injury animal model has also been presented a delayed recovery from SUI after simulated birth injury (5, 26). Dual injury was performed as we described before. In simple terms, Sprague–Dawley rats were anesthetized with ketamine (100 mg/kg) and xylazine (10 mg/kg). For VD, a modified 10F Foley balloon catheter was inserted and stitched into the vagina; the balloon was inflated with 3 ml of water for 4 h. Immediately after VD, the pudendal nerve was identified in ischiorectal space and then isolated and crushed bilaterally with a needle holder twice for 30 s each. Sham birth injury was performed by inserting a catheter without filling the balloon, and opening the ischiorectal fossa but without crushing the nerves. All rats received buprenorphine (0.1 mg/kg) for postoperative analgesia (Figure 1).

### Animals

#### Animals Used for Determination of the MSC Engraftment After Combined Treatment With CCR1-Overexpressing MSCs and CCL7

Forty virgin Sprague–Dawley rats (weight 180–250 g) were evenly distributed into the following four groups (*n* = 10): (1) Sham SUI + MSC-CCR1+CCL7 group, injection of CCR1-overexpressing MSCs intravenously and CCL7 in urethra immediately after sham dual injury; (2) SUI + MSCs group, intravenous injection of MSCs without genetic modification immediately after dual injury; (3) SUI + MSC-CCR1 group, intravenous injection of CCR1-overexpressing MSCs immediately after dual injury, and (4) SUI + MSC-CCR1+CCL7 group, injection of CCR1-overexpressing MSCs intravenously and CCL7 in urethra immediately after dual injury. One week after injection, animals were examined *in vivo* using IVIS Lumina X5 Imaging System (PerkinElmer Inc., Waltham, MA, USA) and then euthanized by intraperitoneal (i.p.) injection of pentobarbital (100 mg/kg). Urethra and vagina were harvested for qualification of CCL7 and immunofluorescence staining of α-actin and green fluorescent protein (GFP).

#### Animal Used for Determination the Histological and Functional Recovery From SUI After Combined Treatment With CCR1-Overexpressing MSCs and CCL7

An additional 30 virgin SD rats (200–260 g) were distributed into the following three groups of 10 mice each: (1) Sham SUI + sham MSC-CCR1+CCL7 treatment group receiving phosphate-buffered saline injection instead of MSCs and CCl7; (2) SUI + sham MSC-CCR1+CCL7 treatment group, and (3) SUI + MSC-CCR1+CCL7 treatment group. The SUI model was induced by dual injury, and MSC-CCR1 and CCL7 were injected as described above. One week after injection, rats were euthanized after the functional tests including EUS EMG, PNMBP, and LPP measurements. Urethra and vagina were harvested for histological examination.

### MSC Preparations

Bone marrow-derived MSCs were obtained from Shanghai Gene Chemical Co., Ltd. (Shanghai, China), and MSCs were isolated from the bone marrow of 2-months-old female Sprague–Dawley rats. MSCs were cultured in α-MEM (Gibco, USA) supplemented with 10% FBS (Gibco, USA) at 37°C in a humidified 5% CO_2_ atmosphere. The medium was replaced every 2 days. MSCs were identified by flow cytometric analysis of surface markers CD34, CD44, CD45, and CD105.

The CCR-1 gene was designed with the target sequence 5′-CTCCGAACACATCTGAACA-3′. A bicistronic expression system consisting of an internal ribosome entry site (IRES) (GeneChem, Shanghai, China) and a GFP (GeneChem, Shanghai, China) was cloned into the murine stem cell virus vector pMSCV gene (GeneChem, Shanghai, China). cDNA for murine CCR1 was inserted in front of IRES-GFP in pMSCV under the control of a constitutively expressed promoter, a specifically designed 5′ long terminal repeat (LTR) from the murine stem cell PCMV virus. This promoter prevents transcriptional suppression and drives high-level constitutive expression of a target gene. The pMSCV-IRES-GFP and the pMSCV-CCR1/IRES-GFP were cloned into TMPT67 cells (DMEM supplemented with 10% FBS). Lentiviruses were produced, harvested, and purified. Cells were transfected with lentivirus by using 6-μg/ml polybrene (Sigma, USA). We assessed successful retroviral transduction by detecting GFP under a fluorescent microscope and selecting stable expression cells for intravenous injection in rats.

### Injection of CCR1 Overexpression MSCs and CCL7

The fluorescent-labeled MSCs/MSC-CCR1 (2 million labeled MSCs suspended in 1 ml of phosphate-buffered saline) was injected into the rats via tail vein (27, 28). Two micrograms of active CCL7 peptide was injected into the periurethral tissues in rats. The sham injection was performed by replacing CCL7 or MSCs or MSC-CCR1 with the same volume of phosphate-buffered saline.

One week after injection, the fluorescent-labeled MSCs were observed *in vivo* using IVIS Lumina X5 Imaging System (PerkinElmer Inc., Waltham, MA, USA). Subsequently, rats were euthanized by intraperitoneal injection of pentobarbital (100 mg/kg). Injured tissues around the pelvic outlet, including urethra and vagina, were harvested for analysis of MSC engraftment using Lumina X5 Imaging System *ex vivo* and then fixed in 4% paraformaldehyde for immunofluorescence staining.

### Immunofluorescent Staining

Eight-micrometer-thick urethral cross-sections were cut and stained for alpha-smooth muscle actin. Tissues were treated with 4% paraformaldehyde, permeabilized with 0.2% Triton-X-100, and then blocked in PBS containing 0.02% bovine serum albumin. Sections were incubated first with a primary antibody against alpha-smooth muscle actin (CST, USA) and then an Alexa 594-conjugated secondary antibody. Slides were washed and mounted with medium containing DAPI (Vector Laboratories). Fluorescent images were obtained using a fluorescence microscope (ECLIPSE Ni-U; Nikon, Tokyo, Japan) with a magnification of ×40 and ×100. GFP-labeled cells were quantified.

### Real-Time Polymerase Chain Reaction (RT-PCR) for CCL7

RT-PCR was performed to evaluate the mRNA expression of CCL7 in urethral tissue in all groups. We extracted total RNA from the middle urethral tissues using Trizol reagent (Invitrogen, USA) according to the manufacturer's instructions. After measurement of RNA concentration, 1 μg of total RNA was reversely transcribed with random primers and the ReverAid First Stand cDNA Synthesis Kit (Thermo Fisher, USA). qRT-PCR was performed using the Power Up SYBR-Green PCR Master Mix kit (Applied Biosystems, USA). For CCL7, the primers were as follows: forward 5′-CTGCCGCGCTTCTGTGT-3′, reverse primers 5′-ACGTGCACGGTGAAAGCA-3′. The gene expression values were normalized using 18s rRNA as a reference gene. All primers used for amplification were purchased from Sangon (Shanghai, China). The relative expression levels were calculated by the 2^−ΔΔCt^ method.

### Urethral Function Testing

Functional testing included external urethral sphincter electromyography (EUS EMG), pudendal nerve potential activity, and leak point pressure (LPP), which were performed as described in our previous studies (5, 26–29). For EUS EMG recording, a parallel platinum bipolar electrode (diameter: 125 mm diameter, Chengyi, Chengdu, China) was inserted into mid-urethra ~5 mm from the bladder neck under stereomicroscope and connected to a recording system after exposing the urethra by pubic symphysectomy. For pudendal nerve potential activity, upon removing the parts of pubis and ischium, pudendal nerve motor branch was identified and separated from the sensory and anal sphincter motor branches with a glass-dissecting needle under a surgical microscope after opening the ischiorectal fossa. The electrodes were connected to an amplifier. A polyethylene catheter (PE 50) was inserted into the bladder transurethrally and connected to a pressure transducer (Chengyi, Chengdu, China) when the bladder was filled with saline (5 ml/h) with a microperfusion pump. LPP testing was performed by filling bladder to half capacity (~0.5 ml) and gentle pressing directly on the bladder until urine leaks from the urethral meatus (5). LPP testing with simultaneous recording of EUS EMG and pudendal nerve potential activity was performed three times in each rat. The bladder was emptied between tests. The bladder was drained if an induced bladder contraction occurred during LPP testing. Mean amplitudes and firing rates of EUS EMG and pudendal nerve potentials activity before and during LPP testing are quantified as previously described (29). The surgical procedures and measurements were performed under urethane anesthesia (1.2 g/kg, intraperitoneal).

### Histology

Immediately after LPP testing, the full-length urethra was collected for histological study. The tissues were immersed and fixed in 4% paraformaldehyde for 24 h and then embedded in paraffin. The cross-section of the mid urethra was sectioned and stained with Masson's trichrome (Solabir, China) for qualitative and semi-quantitative histological analysis. ImageJ 1.52o version Java 1.8.0_112 (NIH, Bethesda, Maryland, USA) was used, and the software can distinguish regions stained with different colors and calculate the areas. The percentages of the muscle (stained in pink) and collagen (stained in blue) area to the whole cross-section area in the middle urethra were calculated.

### Data Analysis and Statistics

Data were expressed as means ± standard error of means (*SE*). Statistical analysis was performed using the SigmaStat 3.5 software (Systat Software, Inc., San Jose, CA). One-way or two-way ANOVA with Tukey's *post-hoc* comparison test or Bonferroni adjustments for multiple comparisons were used to assess statistical significance. A *p* < 0.05 was considered statistically significant.

## Results

### CCR1-Overexpressing MSCs and CCL7 Combined Treatment Increased Engraftment and Survival of MSCs

**Figure 2:**
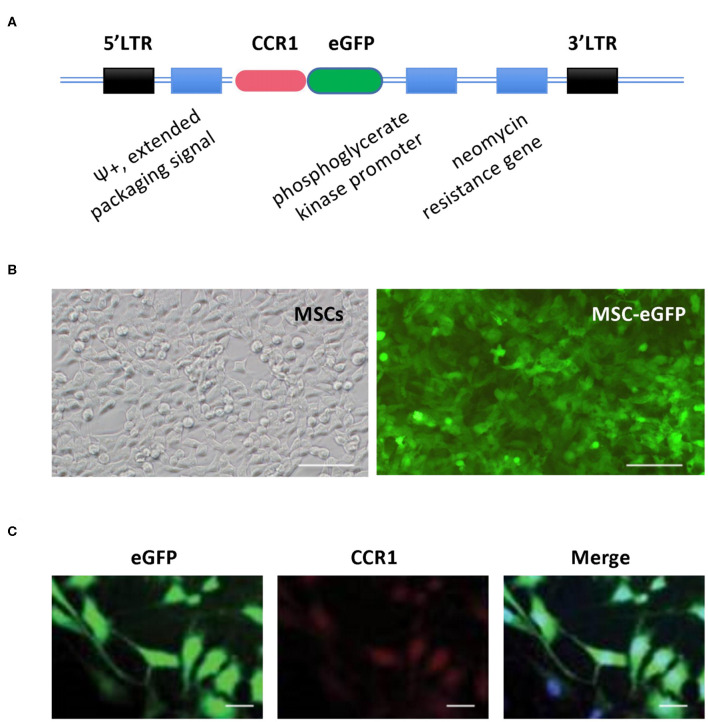
Construction of rat MSCs' vector in expression of CCR1 and eGFP and validation of MSCs-eGFP-CCR1. **(A)** Simplified scheme of the retroviral vector constructs of MSCs-eGFP-CCR1. **(B)** Retrovirus-infected MSCs exhibited robust GFP signal. Phase contrast (left) and green channel (right) images. **(C)** Validation of expression about vector MSCs-eGFP-CCR1. Bar = 50 μm. LTR, long terminal repeats; CCR1, C-C chemokine receptor type 1; eGFP, enhanced green fluorescent protein, MSCs, mesenchymal stem cells.

**Figure 3:**
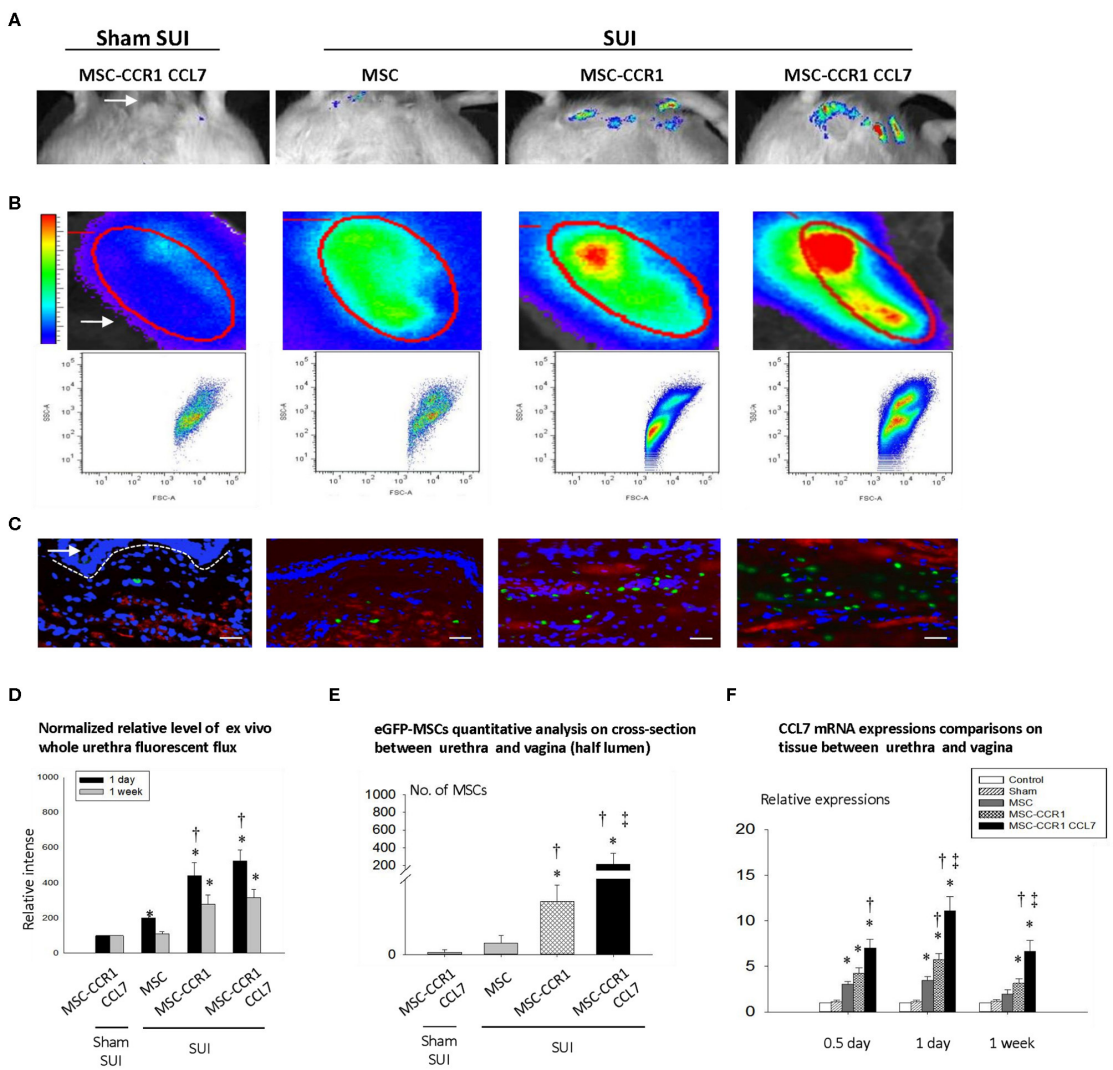
Enhancement of CCL7/CCR1 axis can significantly increase the local engraftment of MSCs in rats with SUI. **(A)** Engraftment of GFP-labeled MSCs in pelvic area one week after injection in different groups. White arrow shows urethral orifice. **(B)** The urethral tissue was taken out, and the fluorescence intensity was measured one week after injection in different groups. White arrow points the urethra *ex vivo*. The elliptical circle shows the analyzed local fluorescence area of the urethra. **(C)** The distribution of MSCs in the urethral cross-sectional area was examined one week after injection, and the dashed line separates the urethral with the vaginal wall, arrows indicates lumen adjacent vaginal anterior wall. Blue staining represents nucleus by DAPI staining; red represents smooth muscle cells (α smooth muscle actin) Green represents eGFP-MSCs. Bar = 100um. **(D)** The relative intensity of fluorescence in isolated urethral tissue at 1 day or 1 week after injection. **(E)** Quantitative analysis of engrafted MSCs in urethra 1 week after injection of MSCs. **(F)** The relative expression of CCL7 in local tissue of urethra, compared with normal urethral tissue by quantitative RT-PCR. * indicates significant difference with the sham injury group (sham SUI, *p* < 0.05); ^†^ indicates significant differences (*P* < 0.05) with normal MSCs before transformation; ‡ indicates significant differences with the modified MSCs-CCR1 (*P* < 0.05). CCL7, chemokine (c-c motif) ligand 7; CCR1, C-C chemokine receptor type 1; MSCs, mesenchymal stem cells; SUI, stress urinary incontinence; DAPI, 4′,6-Diamidino-2-phenyli ndole dihydrochloride; eGFP, enhanced green fluorescent protein; RT-PCR, Real-Time Polymerase Chain Reaction.

To test the potential effect of the receptors in chemokine-induced MSC migration, murine MSCs were retrovirally transfected with CCR1-GFP and the efficiency of the transfection was validated (Figure 2). Cells expressing robust green fluorescence protein were separated and used in this study. MSCs were injected intravenously through the tail vein. *In vivo* (Figure 3A) and *ex vivo* (Figures 3B,D) analysis of the fluorescent-labeled MSCs showed that the SUI + MSC-CCR1+CCL7 group had the strongest green fluorescence signals after 1 week of injury, followed by the groups of SUI + MSC-CCR1, SUI + MSCs, and sham SUI + MSC-CCR1+CCL7 (*p* < 0.05, Figure 3). Moreover, quantifications of the GFP-labeled MSCs in the mid-urethra and the tissue between urethra and vagina also showed that the SUI + MSC-CCR1+CCL7 group had the maximum fluorescent intensity (Figures 3C,E), indicating increased engraftment. Most of the stained cells were located between muscle bundles in the urethra and the tissues between urethra and vagina wall.

We also examined the expression of cytokine CCL7 at 0.5 day, 1 day, and 1 week after simulated birth injury. We found that the expression of CCL7 mRNA in the urethra increased significantly in all three time points, especially in 1 day after dual injury. The SUI + MSC-CCR1+CCL7 group had the highest expression of CCL7 (Figure 3F).

### CCR1-Overexpressing MSCs and CCL7 Combined Treatment Promotes Histological and Functional Recovery

**Figure 4:**
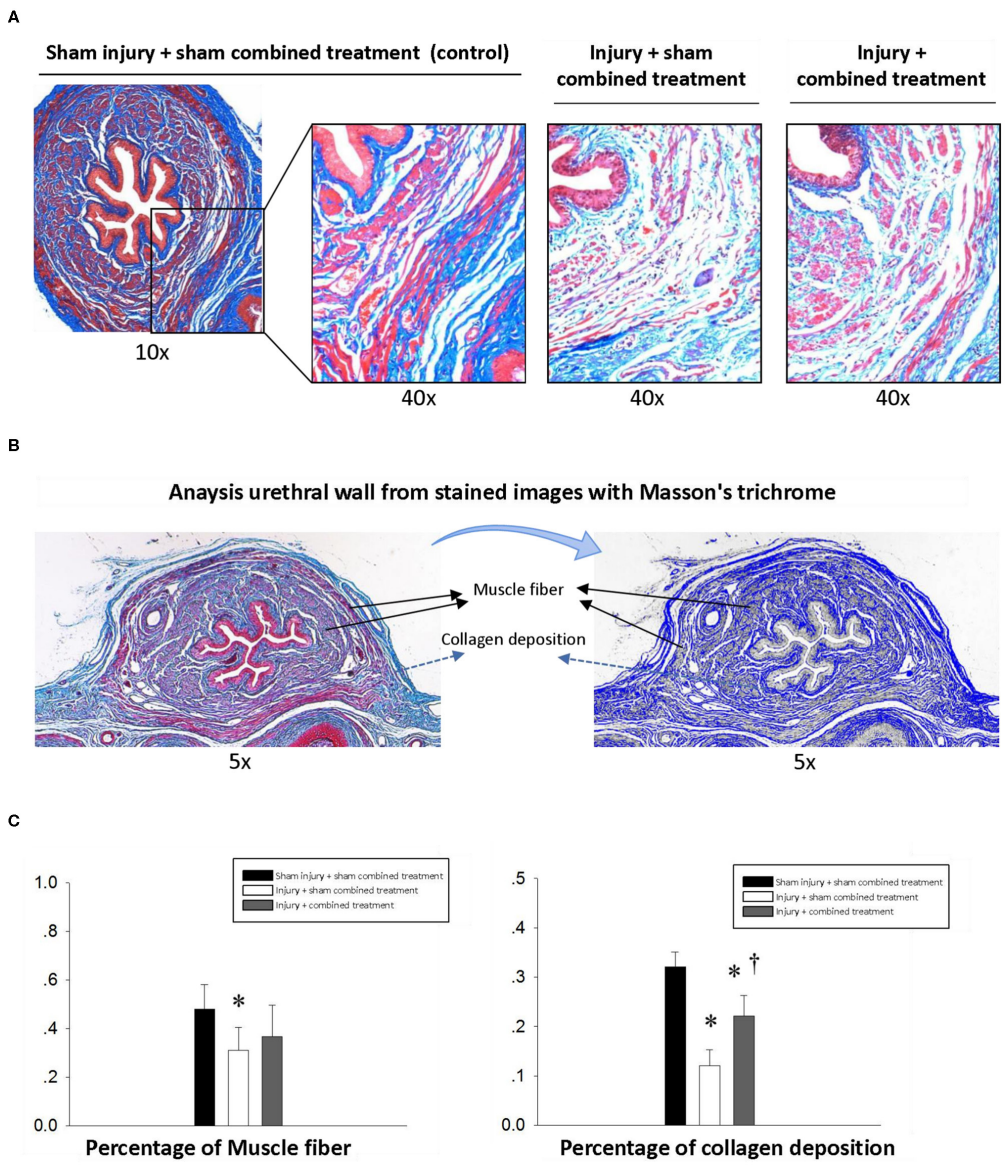
Histological changes of middle urethral cross-sectional area in different groups one week after injection. **(A)** The sphincter atrophy was partially recovered one week after combined treatment. Magnification: 10× and 40×. Stain: Masson's trichrome. **(B)** The middle urethral wall stained with Masson trichrome was used to quantify the area of muscle and collagen. **(C)** Comparison of the percentage of muscle and collagen area in the whole middle urethral wall stained with Masson trichrome among groups. * represents significant difference compared with sham injury + sham combined treatment (*p* < 0.05). ^†^ represents a significant difference compared with injury + sham combined treatment (*p* < 0.05). SUI, stress urinary incontinence; CCR1, C-C chemokine receptor type 1; MSCs, mesenchymal stem cells; CCL7, chemokine (c-c motif) ligand 7.

Simulated birth injury induced by VD and PNC caused damage of the urethra. Masson Trichrome staining of the middle urethra cross-section from the injury (SUI) + sham MSC-CCR1+CCL7 treatment group showed the broken fibers with degeneration and atrophy in the urethral striated muscles 1 week after injury. The muscle fibers shorted, and the space between the muscle fibers was broadened (Figures 4A,B). Quantification data revealed decreased percentage of muscle and collagen area in the whole cross-section area in middle urethra. In the injury (SUI) + MSC-CCR1+CCL7 treatment group, the above changes were partially recovered 1 week after injury, characterized by denser and wider muscle fibers. The percentage of collagen increased significantly, along with the increased percentage of muscle area although the difference was not statistically significant (Figure 4).

**Figure 5:**
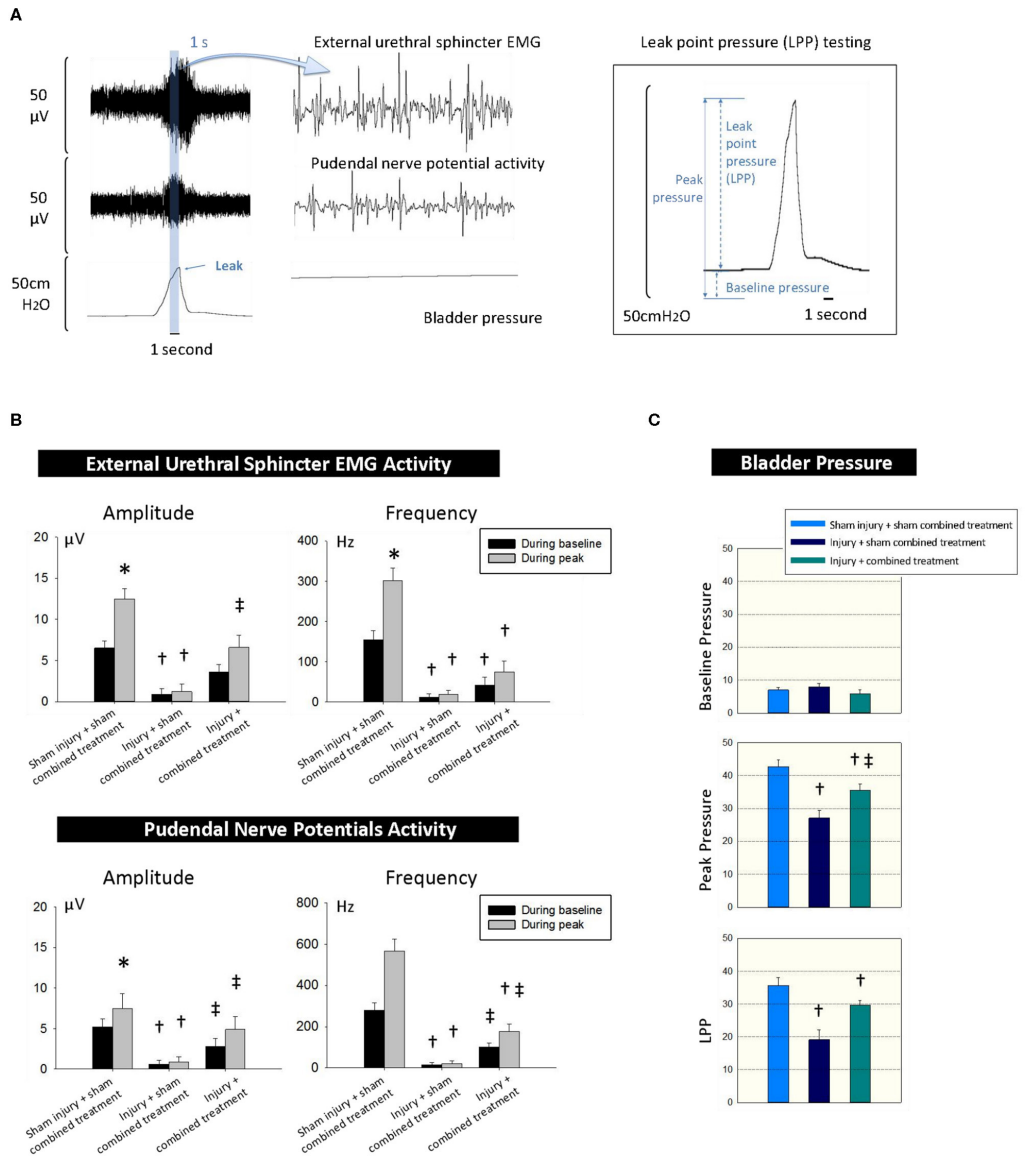
CCR1-overexpressing MSCs and CCL7 combined treatment improves recovery of urinary continence 1 week after injury for SUI. **(A)** The simultaneous measurements of the EUS EMG, pudendal nerve potential activity and bladder pressure for LPP testing 1 week after injury and combined treatment. **(B,C)** Functional recovery differences between the groups of sham injury + sham combined treatment, injury + sham combined treatment, and injury + combined treatment. * represents significant difference compared with baseline (*p* < 0.05), indicating adequate urinary continence level. ^†^ represents a significant difference compared with control (*p* < 0.05), indicating the effect of injury. ‡ represents a significant difference compared with sham combined treatment (*p* < 0.05), indicating the effect of the combined treatment. CCR1, C-C chemokine receptor type 1; MSCs, mesenchymal stem cells; CCL7, chemokine (c-c motif) ligand 7. SUI, stress urinary incontinence; EUS, external urethral sphincter; EMG, electromyography; LPP, leak point pressure.

We simultaneously recorded EUS EMG, PNMBP, and LPP under anesthesia. Both EUS EMG and PNMBP increased significantly in sham SUI + sham MSC-CCR1+CCL7 treatment group when increased pressure was applied to the bladder during an LPP testing, indicating the presence of a guarding response in urethra to the increased bladder pressure. The guarding reflex (increased firing frequency and amplitude of PNMBP or EUS EMG activity) was considered to try to maintain incontinence despite the increase in bladder pressure with the externally applied pressure (Figure 5) (30). Dual injury caused by VD and PNC (injury + sham combined treatment group) led to significantly decreased LPP, along with decreased amplitude (μV) and firing frequency (Hz) in EUS EMG and PNMBP measurement compared with those in sham injury + sham combined treatment group (Figures 5B,C). Combined treatment partially reversed the above changes (Figures 5B,C). LPP, the amplitudes (μV), and frequency of both EUS EMG and PNMBP in injury + MSC-CCR1+CCL7 combined treatment group were significantly higher than those in injury + sham MSC-CCR1+CCL7 combined treatment group (Figures 5B,C), indicating the partially functional recovery.

## Discussion

Vaginal delivery is an important risk factor for the development of SUI. The incidence of SUI in primiparous women was 3.3 times higher than that in nulliparous women (31). Delivery-related injuries are often associated with complex damage, and both nerves and muscles are involved. The endogenous MSCs contribute to the functional recovery and improvement in neurologic outcomes of injured tissues due to their ability of self-renewal and migration to the site of injury (32, 33). With the development of cell-based therapies, MSCs recently gained large attention as a promising treatment for SUI (28, 34, 35). However, the capacity of engraftment and survival of transplanted stem cells into injured urethral tissue remains a major issue (36). A large fraction of intravenous infused MSCs becomes massively trapped within the lung and other organs, which leads to a low engraftment rate to injured tissue (37–39). Multiple processes, including cell recruitment, migration, and adhesion, can affect the incorporation of MSCs into the injured urethral tissue.

Migration and engraftment are a prerequisite for therapeutic MSCs to play their roles in the injured tissue. The mechanisms of MSC engraftment to a target organ have not yet been fully understood. Leukocyte recruitment to inflammatory areas requires a relevant series of cytokines and molecules. This knowledge may help us understand the engraftment of MSCs (40). Previous studies have found that MSCs express a unique set of chemokine receptors that may play an important role in MSC engraftment (41). These findings indicated that a coordinated sequence of interaction between chemokines and chemokine receptors may take effect in MSC engraftment. Therefore, manipulation of the chemokine–chemokine receptor axis may be valuable in MSC-based therapy. Previous studies showed that CC ligands 2, 6, 7, and 9, and CXC ligands 1, 2, and 12 were up-regulated significantly following myocardium ischemia using a functional genomics technology, suggesting that these chemokines may induce the engraftment and aggregation of MSCs (42, 43). Currently, over 50 chemokines and 20 chemokine receptors have been discovered (44). It is one of the prominent features that chemokines can attract MSCs to the injured organs, which has been documented in numerous studies (45–47). Early MSC treatment achieved some success in SUI, but outcomes were not consistent (48, 49). This may be associated with low engraftment and survival rate of the transplanted MSCs. To our knowledge, chemokines inducting MSC migration mainly include CCL3 (formerly known as MIP-1α), CCL4 (formerly known as MIP-1β), CCL5 (formerly known as RANTES), CXCL12 (formerly known as SDF-1), and CCL7 (formerly known as MCP-3) (50, 51). One of the chemokine–chemokine receptor axis, CCL7-CCR1, has been shown to play a crucial role in the recruitment of MSCs into postnatal injured urethral and vaginal tissues (20, 24, 52). A recent study demonstrated that overexpression of CCR1 in MSCs increased their migration and protected them from apoptosis *in vitro*. Intramyocardial injection of CCR1-overexpressing MSCs significantly increased the homing and engraftment to the injured area, prevented cardiac remodeling, and restored cardiac function 4 weeks after myocardial infarction (24). In our current study, we aimed to examine if combined treatment with CCR1-overexpressing MSCs and CCL7 can increase the engraftment and survival of the MSCs and promote the histological and functional recovery.

The engraftment was found in all four groups, including the sham SUI group, indicating that the sham procedure also caused the recruitment of MSCs. Importantly, our data showed that the number of engrafted MSCs in the SUI + MSC-CCR1 and SUI + MSC-CCR1+CCL7 group was significantly higher than the other two groups. The SUI + MSC-CCR1+CCL7 group had the highest engrafted MSCs. Treatment with both chemokine and its receptor can produce synergistic effects. These results demonstrated that overexpression of CCR1 in MSCs promoted their migration and homing, and injection of CCL7 in injured tissues further enhanced the effects. We also found increased expression of CCL7 in the SUI + CCR1-MSCs+CCL7 group after 0.5-day, 1-day, and 1-week injury. The released CCL7 can implement its role via endocrine, autocrine, or paracrine action. Considering the injected CCL7 was already metabolized, the increased CCL7 expression may be because the engrafted MSCs regulate the microenvironment of the injured tissue and promote the release of CCL7.

In addition to increased engraftment, our study also demonstrated that the local injection of CCL7 combined with intravenous delivery of CCR1-overexpressing MSCs can significantly promote structural and functional recovery of the urethra. The amplitude and frequency of EMG and PNMBP were lower significantly in injury (SUI) + sham combined treatment group compared to sham injury + sham combined treatment group, suggesting that the pudendal nerve and urethral muscle were damaged after simulated injury. Partial functional recovery was observed in injury + CCR1-MSCs+CCL7 combined treatment group. Histological results showed a similar trend. The mechanisms of the enhanced therapeutic effects using the combined treatment may be due to (1) increased engraftment and homing of MSCs; (2) increased direct differentiation of MSCs; and (3) promotion repair and regeneration by secreting a range of growth factors, angiogenic factors, and immune regulating factors.

One limitation of our study is that we examined the migration and the aggregation of MSCs and urethral recovery only 1 week after the birth injury. The efficiency of the combined treatment in a longer time needs to be studied in the future.

In summary, in this study, we demonstrated that combined treatment with CCR1-overexpressing MCSs and CCL7 increased the engraftment and survival of MSCs and improved the histological and functional recovery from simulated birth injury-induced SUI in rats. This approach may offer a new strategy for the prevention or treatment of birth trauma-related SUI.

## Data Availability Statement

Part of results were accepted in previous AUA annual meeting. The datasets used and/or analyzed during the current study are available from the corresponding authors on reasonable request.

## Ethics Statement

The animal study was reviewed and approved by The Animal Care Committee at First Affiliated Hospital of Wenzhou Medical University.

## Author Contributions

H-HJ and MW designed the study. GL critically read and revised the manuscript. H-HJ and Q-XS performed the experiments assisted by L-XJ, H-YL, and LC. L-XJ and H-YL assisted H-HJ, MW, and Q-XS with analysis of data. GL and MW interpreted the data. L-XJ and YB drafted the manuscript. All authors have approved the final version of the manuscript and have made substantial contributions to the study. All authors contributed to the article and approved the submitted version.

## Conflict of Interest

The authors declare that the research was conducted in the absence of any commercial or financial relationships that could be construed as a potential conflict of interest.
